# Effect of four premolar extractions on the vertical dimension of the face: A retrospective cephalometric study

**DOI:** 10.1007/s00056-022-00418-2

**Published:** 2022-08-12

**Authors:** Anna Rüedi, Spyridon N. Papageorgiou, Theodore Eliades, Vasiliki Koretsi

**Affiliations:** Clinic of Orthodontics and Pediatric Dentistry, Center of Dental Medicine, University of Zurich, Plattenstrasse 11, 8032 Zurich, Switzerland

**Keywords:** Malocclusion, Bicuspid, Vertical pattern, Fixed appliances, Orthodontics, Malokklusion, Bikuspidale, Vertikales Muster, Festsitzende Apparaturen, Kieferorthopädie

## Abstract

**Purpose:**

Adequate control of the vertical dimension is of great importance in orthodontic treatment. Although existing evidence is very limited, extraction of four premolars is thought to contribute towards improved control of anterior facial height compared with non-extraction treatment protocols. Thus, the aim of this retrospective cohort study was to compare the effect of fixed-appliance treatment with extraction of four premolars to non-extraction treatment on the skeletal vertical dimension.

**Methods:**

A consecutive sample of 76 children with skeletal hyperdivergence (49% male; mean age 11.9 years) was divided into two groups for treatment with either non-extraction (*n* = 31) or extraction of four premolars (*n* = 45). Baseline characteristics were comparable: overjet 5.1 ± 2.5 mm, overbite 2.4 ± 1.9 mm, ANB angle 4.6 ± 2.3°, and SN-ML angle 40.2 ± 3.5°. Patients were treated with standard edgewise fixed appliances with closing loops/sliding mechanics. Vertical skeletal and dental outcomes were measured on lateral cephalograms before and after treatment. Data were analyzed with linear regression at 5%.

**Results:**

Compared to non-extraction treatment, treatment with premolar extractions had no significant effect on the SN-ML angle (difference (Δ) = 0.07°; 95% confidence interval −0.90 to 1.01°; *P* = 0.88). Statistically significant changes between the extraction and non-extraction groups were only found for the parameters SNA (Δ −1.47°; *P* = 0.003), ANB (Δ −1.17°; *P* = 0.004), SN-OP (Δ −1.48°; *P* = 0.04), and L1-ML (Δ −6.39°; *P* < 0.001).

**Conclusion:**

Orthodontic treatment of children with skeletal hyperdivergence using systematic extraction of four premolars had minimal effects on the vertical facial dimension compared to non-extraction treatment.

**Supplementary Information:**

The online version of this article (10.1007/s00056-022-00418-2) contains supplementary material, which is available to authorized users.

## Abstract (de)

**Zielsetzung:**

Eine adäquate Kontrolle der vertikalen Dimension ist in der kieferorthopädischen Behandlung von großer Bedeutung. Auch wenn die Evidenzlage sehr begrenzt ist, geht man davon aus, dass die Extraktion von 4 Prämolaren im Vergleich zu nichtextraktiven Behandlungsprotokollen zu einer besseren Kontrolle der anterioren Gesichtshöhe beiträgt. Ziel dieser retrospektiven Kohortenstudie war es daher, die Auswirkung einer festsitzenden Behandlung mit Extraktion von 4 Prämolaren mit einer nichtextraktiven Behandlung auf die vertikale Dimension des Skeletts zu vergleichen.

**Methoden:**

Eine konsekutive Stichprobe von 76 Kindern mit skelettaler Hyperdivergenz (49% männlich; Durchschnittsalter 11,9 Jahre) wurde in 2 Gruppen aufgeteilt, die entweder mit einer Nichtextraktionsbehandlung (*n* = 31) oder einer Extraktion von 4 Prämolaren (*n* = 45) behandelt wurden. Die Ausgangsdaten waren vergleichbar: Sagittale Frontzahnstufe 5,1 ± 2,5 mm, vertikale Frontzahnstufe 2,4 ± 1,9 mm, ANB-Winkel 4,6 ± 2,3° und SN-ML-Winkel 40,2 ± 3,5°. Die Patienten wurden mit festsitzenden Standardapparaturen mit „closing loops“ /Gleitmechanik behandelt. Die vertikalen skelettalen und die dentalen Resultate wurden auf lateralen Kephalogrammen vor und nach der Behandlung ermittelt. Die Ergebnisse wurden mit einer linearen Regression von 5% ausgewertet.

**Ergebnisse:**

Im Vergleich zu einer Behandlung ohne Extraktion hatte die Behandlung mit Prämolarenextraktionen keine signifikante Auswirkung auf den SN-ML-Winkel (Differenz [Δ] = 0,07°; 95%-Konfidenzintervall [KI] -0,90 bis 1,01°; *p* < 0,88). Statistisch signifikante Veränderungen zwischen der Extraktions- und der Nichtextraktionsgruppe wurden nur für die Parameter SNA (Δ −1,47°; *p* = 0,003), ANB (Δ −1,17°; *p* = 0,004), SN-OP (Δ −1,48°; *p* = 0,04) und L1-ML (Δ −6,39°; *p* < 0,001) festgestellt.

**Fazit:**

Die kieferorthopädische Behandlung von Kindern mit skelettaler Hyperdivergenz durch systematische Extraktion von 4 Prämolaren hatte minimale Auswirkungen auf die vertikale Gesichtsdimension im Vergleich zu einer Behandlung ohne Extraktion.

## Introduction

Controlling the vertical facial dimension during orthodontic treatment is especially important among patients with skeletal hyperdivergence and is often challenging. It is commonly believed that individualized treatment goals and protocols according to the patient’s vertical configuration contribute to improved vertical control. In patients with an increased vertical dimension, this goal would be to minimize molar extrusion, since that would lead to clockwise mandibular rotation [1, 2]. Apart from treatment mechanics, orthodontic treatment including systematic extractions of four premolars has long been thought to result in favorable outcomes in hyperdivergent cases [3].

The bite-closing tendency associated with premolar extractions has been attributed to protraction of the posterior teeth into the extraction sites that might allow the mandible to rotate counterclockwise, thereby reducing the so-called “wedge effect”. Furthermore, reciprocal space closure with retraction and possible retroclination of the anterior teeth could additionally deepen the bite, the so-called “drawbridge effect” [4]. These changes associated with extraction therapy are considered beneficial and could lead to a decrease in lower anterior facial height and a decrease in lip strain [1, 5], thus, improving esthetics in hyperdivergent cases. However, the impact of extraction therapy on the inclination of the mandible and the corresponding reduction in anterior facial height is not unanimously supported in the literature. A systematic review concluded that there is no specific effect of systematic premolar extractions on the skeletal vertical measurements but stated that uncertainty exists in these findings due to the high risk of bias of available studies [6].

Previous studies assessing the effect of extractions on the skeletal vertical dimension included only class II patients thereby limiting generalizability [7–10], did not control for crowding or bimaxillary protrusion [11–13] thereby not allowing for estimation of the “wedge effect”, or did not ensure comparability between extraction and non-extraction groups [7, 14, 15]. Thus, the present study aimed to cephalometrically investigate the effect of four premolar extractions on the vertical dimension in hyperdivergent patients with fixed appliances, irrespective of anteroposterior relationships.

## Materials and methods

This retrospective cohort study was based on the archives of patients treated at the Clinic of Orthodontics and Pediatric Dentistry, University of Zurich, Switzerland. The protocol of the study was developed in advance, but it was not registered, and received ethical approval by the Cantonal Ethics Committee Zurich (BASEC-No.: 2020-02717). All patients or their guardians signed an informed consent prior to their treatment initiation.

Patients’ eligibility was consecutively proved on the basis of the following criteria without considering other factors, such as treatment outcome, duration, patient’s cooperation: (i) available diagnostic records of high quality, (ii) hyperdivergent patients with a SN-ML angle of 34° or more, (iii) no more than 6 mm crowding in the lower dental arch, (iv) patients treated with fixed edgewise appliances with extraction of four premolars for orthodontic purposes or without premolar extractions, (v) no missing teeth or tooth anomalies, (vi) no syndromes or clefts, physical or mental impairment, and (vii) no previous orthodontic treatment.

Sample size calculation was based on a previous study assessing the treatment of patients with skeletal hyperdivergence [10], from which the assumed response for the SN-ML in the non-extraction group was taken (mean 39.3°; standard deviation [SD] 4.5°). Assuming a minimal clinical difference of 10% with the same SD, a total of 62 patients (31 patients per group) would be needed to identify a minimal clinically relevant difference of 10% (with common SD) using an independent-samples t‑test at alpha (α) = 5% and beta (β) = 10%. It was decided to set power to 90%, as previous studies had failed to identify any differences. Therefore, all consecutively eligible patients were included in this study until a minimum of 31 patients per group were included.

All patients were treated by postgraduate students in orthodontics under the direct supervision of experienced clinical instructors. Treatment planning and procedures followed the protocol of the clinic. All present patients received standard edgewise fixed appliances with a 0.018-inch slot. Archwire sequence and treatment mechanics were specified according to each patient’s needs. Either sliding mechanics or closing loops were used for space closure with a 0.016 × 0.022-inch stainless steel wire.

Patient characteristics (sex, age, teeth extracted, overjet, overbite, crowding, orthodontic appliances) were extracted from the patients’ files. Lateral cephalograms were taken in natural head position at two timepoints: prior to treatment initiation (T1) and after debonding (T2). They were traced to assess several skeletal and dental parameters (Supplementary Table 1). The primary outcome of this study was the divergence of the skeletal mandibular base (SN-ML angle). To assess consistency of the measurement method, a set of 50 lateral cephalograms was remeasured by the first author within a 2-week interval and another set of 50 lateral cephalograms was remeasured by the last author.

Although all material for this study was coded, no blinding was possible at outcome measurement, since extraction treatment can easily be identified on lateral cephalograms. However, statistical analysis was undertaken blindly using a coded dataset.

### Statistical analyses

Initially, descriptive statistics were calculated after checking normality, including means and SDs for normally distributed continuous outcomes (medians and interquartile ranges [IQR] for skewed data) or absolute/relative frequencies for categorical outcomes. Differences in baseline characteristics were assessed with independent-samples t‑tests, Mann–Whitney tests, or Χ^2^ tests. Crude differences in outcomes were assessed with linear regression using T2 values as dependent variables, the extraction group as independent variable and T1 values as covariates. Adjusted regressions were run to control for potential confounders, which were selected with a 10% change-in-estimate approach. All analyses were run in StataSE 13.0 (StataCorp, College Station, TX, USA) with a two-sided α of 5% and an open dataset available through Zenodo [16].

## Results

In all, 76 patients, of which 51% (*n* = 39) were females, with a mean age of 11.9 ± 1.7 years, were included in the present study (Table 1). From these patients, 31 were treated with four premolar extractions and 45 were treated non-extraction. No significant differences (*P* > 0.05) in patients’ age, sex, overjet, or overbite were observed at baseline. The average overjet was 5.1 ± 2.5 mm and the average overbite was 2.4 ± 1.9 mm. The only difference was the available arch space, which in the extraction group was significantly less than in the non-extraction group for both the upper (−2.1 versus −0.7 mm) and the lower (−2.0 versus +0.7 mm) arches.

**Table 1:** Demographics of included patients Demografische Details der aufgenommenen Patienten

Variable	Metric	Overall (*n* = 76)	Non-Ex (*n* = 31)	Ex (*n* = 45)	*P*
Age (years)	Mean (SD)	11.9 (1.7)	11.7 (1.8)	12.1 (1.6)	0.36^a^
Sex	Male, *n* (%)	37 (49%)	17 (55%)	20 (44%)	0.37^b^
	Female, *n* (%)	39 (51%)	14 (45%)	25 (56%)	
Overjet (mm)	Mean (SD)	5.1 (2.5)	5.1 (2.8)	5.0 (2.4)	0.82^a^
Overbite (mm)	Mean (SD)	2.4 (1.9)	2.6 (2.1)	2.3 (1.8)	0.48^a^
Upper space (mm)	Median (IQR)	−1.3 (−3.3, 0.8)	−0.7 (−1.9, 2.5)	−2.1 (−4.0, −0.5)	*0.006*^c^
Lower space (mm)	Mean (SD)	−0.9 (2.8)	0.7 (2.5)	−2.0 (2.4)	*<0.001*^a^
Extracted teeth	4 × 4 s, *n* (%)	–	–	5 (12%)	–
	2 × 5 s, 2 × 4 s, *n* (%)	–	–	23 (53%)	
	4 × 5 s, *n* (%)	–	–	15 (35%)	
Treatment time (months)	Mean (SD)	30.1 (11.6)	27.5 (12.0)	32.0 (11.1)	0.10^a^

*IQR* interquartile range, *SD* standard deviation, *Non-ex* non-extraction, *Ex* extraction of 4 premolars

^a^t‑test for independent samples

^b^Χ^2^ test

^c^Mann–Whitney test

Cephalometrically, the two groups were very similar for most skeletal and dental parameters (Table 2). The included sample showed an increased vertical dimension with a mean SN-ML of 40.2° (SD 3.5°), SN-NL of 6.8° (SD 3.7°), and SN-OP of 20.4° (SD 3.3°) and a class II tendency with an ANB of 4.6° (SD 2.3°). The only significant differences between groups at baseline were seen for NL-ML (34.5° and 31.9° in the extraction and non-extraction groups, respectively) and SpaMe:NMe (59.1% and 58.0% in the extraction and non-extraction groups, respectively). The inclinations of the upper and lower incisors were similar between the groups and normal at baseline with U1-NL and L1-ML of 109.8° (SD 7.2°) and 91.9° (SD 7.3°), respectively.

**Table 2:** Baseline cephalometric assessment of included patients Kephalometrische Ausgangsuntersuchung der aufgenommenen Patienten

Variable	Metric	Overall (*n* = 76)	Non-Ex (*n* = 31)	Ex (*n* = 45)	*P*^a^
SNA (°)	Mean (SD)	79.8 (3.4)	79.1 (3.5)	80.2 (3.2)	0.15
SNB (°)	Mean (SD)	75.2 (3.0)	74.7 (3.6)	75.5 (2.5)	0.22
ANB (°)	Mean (SD)	4.6 (2.3)	4.4 (2.2)	4.7 (2.3)	0.59
ArGoMe (°)	Mean (SD)	129.7 (5.8)	128.7 (5.5)	130.5 (6.0)	0.18
SN-ML (°)	Mean (SD)	40.2 (3.5)	39.6 (3.1)	40.7 (3.7)	0.18
SN-NL (°)	Mean (SD)	6.8 (3.7)	7.7 (4.8)	6.2 (2.5)	0.08
NL-ML (°)	Mean (SD)	33.4 (3.9)	31.9 (3.7)	34.5 (3.7)	*0.004*
SN-OP (°)	Mean (SD)	20.4 (3.3)	20.8 (3.5)	20.1 (3.2)	0.39
SAr:ArGo (%)	Mean (SD)	83.1 (9.8)	82.5 (9.2)	83.6 (10.3)	0.61
SGo:NMe (%)	Mean (SD)	58.9 (2.8)	59.1 (2.6)	58.8 (2.9)	0.68
SpaMe:NMe (%)	Mean (SD)	58.7 (2.2)	58.0 (2.4)	59.1 (2.0)	*0.04*
U1-NL (°)	Mean (SD)	109.8 (7.2)	110.0 (8.3)	109.6 (6.5)	0.84
L1-ML (°)	Mean (SD)	91.9 (7.3)	92.6 (7.1)	91.5 (7.5)	0.51

*IQR* interquartile range, *SD* standard deviation,* Non-ex* non-extraction, *Ex* extraction of 4 premolars

^a^From t‑test for independent samples

As far as treatment appliances are concerned, the only statistically significant difference between the groups was the use of a lingual arch in 13% of the patients in the non-extraction group and 44% of the patients in the extraction group (Supplementary Table 2).

Orthodontic treatment with extraction of four premolars had little effect on the vertical dimension (Table 3). Changes in mandibular divergence (SN-ML) were similar in the non-extraction (−0.6°) and extraction (−0.5°) groups, to a difference in means of 0.1° (95% confidence interval [CI] = −0.9 to 1.1°; *P* = 0.88). Similarly, no significant differences were found for SN-NL (0.8° and 0.9° in the non-extraction and extraction groups, respectively) or for NL-ML (−1.3° and −1.4° in the non-extraction and extraction groups, respectively). The only difference was observed for the SN-OP with significantly less anterior rotation in the non-extraction group (−0.2°) compared to the extraction group (−1.5°), resulting in a difference in means of −1.5° (95% CI −2.9 to −0.1°; *P* = 0.04). Furthermore, no significant differences were found for SAr:ArGo (*P* = 0.88), SGo:NMe (*P* = 0.47), or SpaMe:NMe (*P* = 0.29).

**Table 3:** Cephalometric changes during treatment in the non-extraction and extraction groups Kephalometrische Veränderungen während der Behandlung in der Nichtextraktions- und in der Extraktionsgruppe

Variable	Group	T1 Mean (SD)	T2 Mean (SD)	T2–T1 change	% change	*P*^a^
SNA (°)	Non-Ex	79.1 (3.5)	79.1 (3.9)	0	0	*0.003*
	Ex	80.2 (3.2)	78.7 (3.8)	−1.5	−1.9	
SNB (°)	Non-Ex	74.7 (3.6)	75.0 (3.9)	+0.3	+0.4	0.51
	Ex	75.5 (2.5)	75.6 (3.1)	+0.1	+0.1	
ANB (°)	Non-Ex	4.4 (2.2)	4.1 (1.9)	−0.4	+117	*0.004*
	Ex	4.7 (2.3)	3.1 (2.3)	−1.6	−149	
ArGoMe (°)	Non-Ex	128.7 (5.5)	126.8 (5.6)	−1.8	−1.4	0.12
	Ex	130.5 (6.0)	129.4 (5.9)	−1.0	−0.8	
SN-ML (°)	Non-Ex	39.6 (3.1)	39.0 (4.1)	−0.6	−1.5	0.88
	Ex	40.7 (3.7)	40.2 (4.1)	−0.5	−1.1	
SN-OP (°)	Non-Ex	20.8 (3.5)	20.6 (3.7)	−0.2	+0.3	*0.04*
	Ex	20.1 (3.2)	18.6 (3.7)	−1.5	−6.7	
SN-NL (°)	Non-Ex	7.7 (4.8)	8.5 (4.7)	+0.8	+34.1	0.67
	Ex	6.2 (2.5)	7.2 (2.6)	+0.9	+16.4	
NL-ML (°)	Non-Ex	31.9 (3.7)	30.6 (4.8)	−1.3	−4.0	0.53
	Ex	34.5 (3.7)	33.1 (3.7)	−1.4	−3.6	
SAr:ArGo (%)	Non-Ex	82.5 (9.2)	79.4 (10.6)	−3.1	−3.7	0.88
	Ex	83.6 (10.3)	80.6 (9.9)	−3.0	−3.4	
SGo:NMe (%)	Non-Ex	59.1 (2.6)	60.0 (3.0)	+0.9	+1.6	0.47
	Ex	58.8 (2.9)	59.5 (3.2)	+0.7	+1.2	
SpaMe:NMe (%)	Non-Ex	58.0 (2.4)	58.3 (2.7)	+0.2	+0.4	0.29
	Ex	59.1 (2.0)	58.6 (2.0)	−0.5	−0.9	
U1-NL (°)	Non-Ex	110 (8.3)	108.1 (6.9)	−1.9	−1.2	0.23
	Ex	109.6 (6.5)	106.4 (5.5)	−3.2	−2.6	
L1-ML (°)	Non-Ex	92.6 (7.1)	96.1 (5.2)	+3.5	+4.3	*<0.001*
	Ex	91.5 (7.5)	89.2 (7.4)	−2.2	−2.2	

*IQR* interquartile range, *SD* standard deviation, *Non-ex* non-extraction, *Ex* extraction of 4 premolars

^a^From linear regression with T2 value as outcome, extraction group as independent variable and T1 value as covariate

As far as sagittal skeletal parameters are concerned, the extraction group presented a greater reduction (−1.5°) than the non-extraction group (0°; *P* = 0.003) in the SNA angle, as well as a greater reduction (−1.6°) than the non-extraction group (−0.4°; *P* = 0.004) in the ANB angle.

Incisor inclination was only slightly affected by the decision to extract four premolars or not. The upper incisors were slightly more retroclined in the extraction group (−3.2°) than in the non-extraction group (−1.9°), which was not statistically significant. On the other hand, significant differences were observed for the inclination of the lower incisors, which were proclined in the non-extraction group (+3.5°) and slightly retroclined in the extraction group (−2.2°).

No significant effects from confounders (Supplementary Table 3) were noted for the primary outcome (SN-ML) or the two selected secondary outcomes (SGo-NMe, SpaMe:NMe; Table 4). The effect of premolar extractions on both SN-ML and SGo:NMe was nonsignificant in the crude analysis and remained so after adjusting for confounders. The effect of extractions on SpaMe:NMe was nonsignificant on average in the crude analysis (−0.44°), but hints of confounding according to the specific premolars extracted were identified. Different effects on SpaMe:NMe were observed for the extraction of four first premolars (difference (Δ) −1.39% to the non-extraction group), upper first/lower second premolars (Δ −0.86% to the non-extraction group), or four second premolars (Δ 0.47% to the non-extraction group), with statistically significant differences among them (*P* = 0.04).

**Table 4:** Effect of four premolar extractions on the vertical parameters at T2 (with the vertical parameter at T1 as covariate) using either crude models or models adjusted for selected baseline covariates (in Supplementary Table 3) Auswirkung der Extraktion von 4 Prämolaren auf die vertikalen Parameter bei T2 (mit dem vertikalen Parameter bei T1 als Kovariable) unter Verwendung von Rohmodellen bzw. für ausgewählte Ausgangskovariablen angepasste Modellen (vgl. ergänzende Tab. 3)

	SN-ML		SGo:NMe		SpaMe:NMe	
Variable	β (95% CI)	*P*	β (95% CI)	*P*	β (95% CI)	*P*
Nothing	0.07 (−0.90, 1.05)	0.88	−0.28 (−1.06, 0.49)	0.47	−0.44 (−1.27, 0.39)	0.29
Age at T1	−0.01 (−0.98, 0.97)	0.99	−0.22 (−1.00, 0.55)	0.57	−0.58 (−1.37, 0.21)	0.15
Sex	0.06 (−0.92, 1.05)	0.90	NT	NT	−0.50 (−1.33, 0.33)	0.23
Overjet at T1	NT	NT	NT	NT	NT	NT
Overbite at T1	−0.23 (−1.12, 0.67)	0.61	−0.08 (−0.80, 0.65)	0.84	NT	NT
Upper space at T1	0.01 (−1.03, 1.04)	0.99	−0.34 (−1.16, 0.48)	0.41	−0.17 (−1.01, 0.67)	0.69
Lower space at T1	−0.31 (−1.40, 0.79)	0.58	−0.11 (−1.00, 0.79)	0.82	−0.72 (−1.66, 0.21)	0.13
SNA at T1	0.14 (−0.88, 1.15)	0.79	NT	NT	NT	NT
SNB T1	0.10 (−0.92, 1.12)	0.85	NT	NT	NT	NT
ANB at T1	0.08 (−0.90, 1.07)	0.86	NT	NT	NT	NT
ArGoMe at T1	NT	NT	−0.23 (−1.01, 0.55)	0.56	NT	NT
Extracted teeth	0.39 (−0.96, 1.73)	0.57	−0.03 (−2.01, 1.96)	0.98	−2.81 (−4.75, −0.87)	*0.005*
Headgear	0.04 (−0.95, 1.02)	0.94	−0.25 (−1.03, 0.54)	0.53	−0.35 (−1.20, 0.49)	0.41
Rapid maxillary expansion	0.13 (−0.85, 1.11)	0.79	NT	NT	−0.59 (−1.39, 0.21)	0.14
Lingual arch	−0.03 (−1.07, 1.00)	0.95	−0.23 (−1.06, 0.59)	0.58	NT	NT

*CI* confidence interval, *NT* not tested, *β* unstandardized regression coefficient

For all cephalometric measurements, concordance was almost perfect within examiner (range 0.9–0.996) and between examiners (range 0.925–0.997). Systematic bias was also extremely low both within and between examiners (up to 0.35° for all cephalometric angles and 0.65% for the cephalometric ratios; Supplementary Table 4).

## Discussion

This study assessed the effect of systematic premolar extractions on the vertical skeletal dimension in 76 children with skeletal hyperdivergence treated with fixed appliances. Extraction of four premolars had no consistent effect on the skeletal vertical dimension of the face represented by the cephalometric measurements SN-ML, SpaMe:NMe, and SGo:NMe. The only significant influences pertained to dental parameters like SN-OP and L1-ML, where that the extraction group presented an increased anterior rotation of the occlusal plane and an increased retroclination of the lower incisors. Furthermore, the SNA and, subsequently, ANB angles were decreased in the extraction group (Table 3), but this could still be due to treatment-induced alterations to point A [17]. Apart from the decision which teeth to be extracted on the variable SpaMe:NMe, none of the covariates (dental or skeletal parameters as well as orthodontic appliances) identified (Supplementary Table 3) and tested (Table 4) had a considerable confounding effect on any of the skeletal vertical cephalometric parameters.

According to the results of the present study, it seems that the effect of four premolar extraction therapy on the vertical skeletal dimension is not apparent in preadolescent and adolescent children with skeletal hyperdivergence. On the contrary, in a similar hyperdivergent class II sample, Porto et al. [18] reported a statistically significant decrease in the SN-GoGn angle and facial axis (SN-Gn angle) of less than 1° in the extraction group compared with an increase of less than 1.5° in the non-extraction group. A similar beneficial effect on the anterior facial height was also noted for the extraction group compared to the non-extraction group in both class I and II patients [5]. Furthermore, in a slightly older borderline sample selected with discriminant analysis based on parameters relating to the extraction decision, significantly different effects (*P* = 0.04) on the vertical dimension were seen according to premolar extractions: a small reduction in the SN-GoGn angle was found in the extraction group, but a small increase in the non-extraction group [19].

Even if statistically significant, the effect of four premolar extraction treatment on the vertical cephalometric variables seems to be of questionable clinical importance due to its small magnitude, which falls within the range of systematic error or small baseline differences. Also, data from many studies indicate that treatment with four premolar extractions does not actually affect the vertical skeletal pattern [20–26]. It seems that the majority of evidence in support of extraction treatment to control or reduce the vertical dimension originates from older publications, which did not directly investigate the effect of extractions and/or were case reports or opinion papers [4, 27, 28].

With respect to the decision of which teeth to be extracted, only the extraction of four second premolars or molars led to a counterclockwise mandibular rotation, while extraction of four first premolars had no significant treatment effect on the mandible in a prospective investigation of 15 years olds with skeletal open bite [29]. On the other hand, no difference in the vertical facial dimension could be identified between first and second premolar extraction treatment in patients with skeletal hyperdivergence in other studies [30, 31]. On the contrary, in the present study, compared to non-extraction therapy, a slight decrease in the anterior facial height ratio was observed only for the extraction of four first premolars, but not for the extraction of four second premolars. It should be noted, however, that in the present study, 53% of patients in the extraction group was treated with two extractions of the first and two extractions of the second premolars. Since the sample was collected irrespective of anteroposterior discrepancies, different anchorage needs in the sagittal dimension most probably might have dictated which teeth to be extracted.

The strengths of the present study include the a priori study design with hypotheses defined in advance to ensure appropriateness of the study population, selected outcomes, and covariates [32]. Furthermore, the sample size was calculated in advance to ensure enough power and prevent inflated results. The variety of orthodontists and clinical instructors can enhance generalizability of the present results. Finally, blinding at the statistical and interpretation level could reduce potential bias and the openly available dataset increases transparency. On the other hand, this study certainly presents some limitations primarily relating to the retrospective study design. First, retrospective nonrandomized studies have been shown to present inflated intervention effects in orthodontic research [33]. Second, apart from the general treatment protocol of the clinic, each patient’s treatment was carried out in accordance with each patient’s needs. Although treatment appliances were controlled for their effect in this study, a prospective study design could have better ensured standardization of treatment procedures. In that context, the only statistically significant difference between the groups regarding treatment appliances was the lingual arch, which was used more in the extraction than non-extraction group and might have prevented molars from tipping into the extraction sites [34].

## Conclusions

In children with skeletal hyperdivergence, minimal to moderate crowding, and normal incisor inclinations, it seems that orthodontic treatment with extraction of four premolars is not necessarily associated with improved facial height compared with non-extraction treatment.

### Supplementary Information

Supplementary Tables 1–4
