# Supplementary material for: Effect of four premolar extractions on the vertical dimension of the face: A retrospective cephalometric study
Source: J Orofac Orthop. 2022 Aug 12;85(3):181–8. doi: 10.1007/s00056-022-00418-2 (PMC11035388; doi:10.1007/s00056-022-00418-2)
Supplement: Supplementary file 1 — Supplementary Tables 1–4 [file 56_2022_418_MOESM1_ESM.pdf]

Supplementary Table 1 Cephalometric measurements used in this study along with their definitions

**Variable**	**Definition**
SNA	The angle formed by the anterior cranial base (Sella to Nasion) and landmark A point
SNB	The angle formed by the anterior cranial base (Sella to Nasion) and landmark B point
ANB	The angle formed by the landmarks A point, B point, and Nasion
ArGoMe	The gonial angle, formed by the landmarks Articulare, Gonion, and Menton
SN-ML	The angle formed by the anterior cranial base (Sella to Nasion) and the mandibular plane (Gonion to Menton)
SN-NL	The angle formed by the anterior cranial base (Sella to Nasion) and the maxillary plane (anterior nasal spine to posterior nasal spine)
NL-ML	The angle formed by the maxillary plane (anterior nasal spine to posterior nasal spine) and the mandibular plane (Gonion to Menton)
SN-OP	The angle formed by the anterior cranial base (Sella to Nasion) and the occlusal plane (distal cusp of the first molars to a point corresponding to half the overbite)
SAr:ArGo	The ratio between the lines connecting the landmarks Sella to Articulare and Articulare to Gonion
SGo:NMe	The ratio between the lines connecting the landmarks Sella to Gonion and Nasion to Menton
SpaMe:NMe	The ratio between the lines connecting the landmarks anterior nasal spine to Menton and Nasion to Menton
U1-NL	The angle formed by the long axis of the maxillary incisors and the maxillary plane (anterior nasal spine to posterior nasal spine)
L1-ML	The angle formed by the long axis of the mandibular incisors and the mandibular plane (Gonion to Menton)

Supplementary Table 2 Treatment appliances for the included sample

**Variable**	**Metric**	**Overall (n=76)**	**Non-Ex (n=31)**	**Ex (n=45)**	***P***
Headgear	No – n(%)	27 (35%)	9 (29%)	18 (40%)	0.33[a]
	Yes – n(%)	49 (65%)	22 (71%)	27 (60%)	
Nance appliance	No – n(%)	71 (93%)	31 (100%)	40 (89%)	0.08[b]
	Yes – n(%)	5 (7%)	0 (0%)	5 (11%)	
Lipbumper	No – n(%)	73 (96%)	29 (94%)	44 (98%)	0.56[b]
	Yes – n(%)	3 (4%)	2 (6%)	1 (2%)	
Monoblock-Headgear	No – n(%)	72 (95%)	29 (94%)	43 (96%)	1.00[b]
	Yes – n(%)	4 (5%)	2 (6%)	2 (4%)	
Monoblock	No – n(%)	72 (95%)	29 (94%)	43 (96%)	1.00[b]
	Yes – n(%)	4 (5%)	2 (6%)	2 (4%)	
Facemask	No – n(%)	75 (99%)	31 (100%)	44 (98%)	1.00[b]
	Yes – n(%)	1 (1%)	0 (0%)	1 (2%)	
Rapid maxillary expansion	No – n(%)	63 (83%)	24 (77%)	39 (87%)	0.29[a]
	Yes – n(%)	13 (17%)	7 (23%)	6 (13%)	
E-arch	No – n(%)	75 (99%)	30 (97%)	45 (100%)	0.41[b]
	Yes – n(%)	1 (1%)	1 (3%)	0 (0%)	
W-arch	No – n(%)	75 (99%)	30 (97%)	45 (100%)	0.41[b]
	Yes – n(%)	1 (1%)	1 (3%)	0 (0%)	
Transpalatal arch	No – n(%)	50 (66%)	23 (74%)	27 (60%)	0.20[a]
	Yes – n(%)	26 (34%)	8 (26%)	18 (40%)	
Lingual arch	No – n(%)	52 (68%)	27 (87%)	25 (56%)	**0.005**[b]
	Yes – n(%)	24 (32%)	4 (13%)	20 (44%)	
Plate	No – n(%)	75 (99%)	30 (97%)	45 (100%)	0.41[b]
	Yes – n(%)	1 (1%)	1 (3%)	0 (0%)	

[a] from chi-squared test

[b] from Fisher’s exact test

Supplementary Table 3 Selection of covariates to be entered in adjusted models using the change-in-estimate method with a threshold of 10%

	**SN-ML**		**SGo:NMe**		**SpaMe:NMe**	
**Variable**	**β for Ex**	**% change**	**β for Ex**	**% change**	**β for Ex**	**% change**
Nothing	0.0738	Reference	-0.2847	Reference	-0.4418	Reference
Age	-0.0077	**>100%**	-0.2221	**-22%**	-0.5787	**31%**
Sex	0.0638	**14%**	-0.2677	-6%	-0.5024	**14%**
Overjet at T1	0.0711	4%	-0.2814	-1%	-0.4362	-1%
Overbite at T1	-0.2276	**>100%**	-0.0751	**-74%**	-0.4197	-5%
Upper space at T1	0.0057	**92%**	-0.3398	**19%**	-0.1707	**-61%**
Lower space at T1	-0.3051	**>100%**	-0.1050	**-63%**	-0.7214	**63%**
SNA	0.1381	**87%**	-0.2936	3%	-0.4572	3%
SNB	0.0960	**30%**	-0.2850	0%	-0.4455	1%
ANB	0.0846	**15%**	-0.2876	1%	-0.4387	-1%
ArGoMe	0.0721	2%	-0.2306	**-19%**	-0.4411	0%
Extracted teeth	0.3868	**>100%**	-0.0254	**-91%**	-2.8096	**>100%**
Headgear	0.0363	**51%**	-0.2473	**-13%**	-0.3536	**-20%**
Rapid maxillary expansion	0.1297	**76%**	-0.2942	3%	-0.5941	**34%**
Lingual arch	-0.3484	**>100%**	-0.2316	**19%**	-0.4625	-5%

Supplementary Table 4 Evaluation of repeatability and agreement of duplicate intra- and inter-examiner measurements

**Variable**	**Assessment**	**CCC (95% CI)**	**Difference (95% LoA)**	***P*[a]**
SNA	Intra-examiner	0.991 (0.987, 0.996)	-0.029 (-0.998, 0.941)	0.75
SNB	Intra-examiner	0.990 (0.984, 0.996)	-0.025 (-1.064, 1.015)	0.84
ANB	Intra-examiner	0.986 (0.978, 0.994)	-0.004 (-0.792, 0.784)	0.91
ArGoMe	Intra-examiner	0.992 (0.987, 0.996)	-0.063 (-1.616, 1.491)	0.42
SN-ML	Intra-examiner	0.992 (0.987, 0.996)	0.106 (-0.948, 1.161)	0.38
SN-NL	Intra-examiner	0.991 (0.986, 0.996)	0.158 (-0.887, 1.203)	**0.04**
NL-ML	Intra-examiner	0.991 (0.985, 0.996)	-0.051 (-1.126, 1.023)	0.51
SN-OP	Intra-examiner	0.990 (0.984, 0.996)	-0.026 (-1.185, 1.134)	0.94
SAr:ArGo (%)	Intra-examiner	0.986 (0.979, 0.994)	-0.640 (-3.597, 2.317)	**0.003**
SGo:NMe (%)	Intra-examiner	0.970 (0.954, 0.986)	-0.060 (-1.805, 1.685)	0.40
SpaMe:NMe (%)	Intra-examiner	0.900 (0.846, 0.954)	-0.040 (-1.853, 1.773)	0.94
U1-NL	Intra-examiner	0.996 (0.994, 0.998)	0.021 (-1.262, 1.303)	0.21
L1-ML	Intra-examiner	0.996 (0.994, 0.998)	0.186 (-1.142, 1.514)	0.09
SNA	Inter-examiner	0.991 (0.986, 0.996)	-0.159 (-1.282, 0.963)	0.13
SNB	Inter-examiner	0.990 (0.984, 0.995)	-0.327 (-1.114, 0.459)	**<0.001**
ANB	Inter-examiner	0.979 (0.967, 0.991)	0.168 (-0.698, 1.034)	**0.03**
ArGoMe	Inter-examiner	0.997 (0.995, 0.999)	0.037 (-0.916, 0.991)	0.79
SN-ML	Inter-examiner	0.990 (0.985, 0.996)	-0.075 (-1.105, 0.954)	0.39
SN-NL	Inter-examiner	0.990 (0.985, 0.996)	-0.035 (-1.005, 0.935)	0.12
NL-ML	Inter-examiner	0.993 (0.990, 0.997)	-0.040 (-0.989, 0.908)	0.84
SN-OP	Inter-examiner	0.981 (0.971, 0.992)	0.233 (-1.044, 1.510)	**0.05**
SAr:ArGo (%)	Inter-examiner	0.992 (0.987, 0.996)	-0.040 (-2.782, 2.702)	0.43
SGo:NMe (%)	Inter-examiner	0.966 (0.947, 0.985)	0.180 (-1.338, 1.698)	0.27
SpaMe:NMe (%)	Inter-examiner	0.925 (0.884, 0.965)	-0.280 (-1.866, 1.306)	0.06
U1-NL	Inter-examiner	0.997 (0.995, 0.999)	-0.014 (-1.093, 1.065)	0.94
L1-ML	Inter-examiner	0.995 (0.992, 0.998)	0.093 (-1.278, 1.464)	0.58

CCC concordance correlation coefficient, *CI* confidence interval, *LoA* limits of agreement

[a] from Bradley-Blackwood F test
